# Blood-Brain Barrier Disruption in Neuro-Oncology: Strategies, Failures, and Challenges to Overcome

**DOI:** 10.3389/fonc.2020.563840

**Published:** 2020-09-18

**Authors:** Brij S. Karmur, Justine Philteos, Aram Abbasian, Brad E. Zacharia, Nir Lipsman, Victor Levin, Stuart Grossman, Alireza Mansouri

**Affiliations:** ^1^Faculty of Medicine, University of Toronto, Toronto, ON, Canada; ^2^Department of Physiology, University of Toronto, Toronto, ON, Canada; ^3^Penn State Health Neurosurgery, College of Medicine, Penn State University, Hershey, PA, United States; ^4^Division of Neurosurgery, University of Toronto, Toronto, ON, Canada; ^5^Department of Neurosurgery, Medical School, University of California, San Francisco, San Francisco, CA, United States; ^6^Department of Oncology, Johns Hopkins Medicine, Baltimore, MD, United States

**Keywords:** blood-brain barrier, neuro-oncolgy, neurosurgical oncology, clinical trial, drug delivery, glioma

## Abstract

The blood-brain barrier (BBB) presents a formidable challenge in the development of effective therapeutics in neuro-oncology. This has fueled several decades of efforts to develop strategies for disrupting the BBB, but progress has not been satisfactory. As such, numerous drug- and device-based methods are currently being investigated in humans. Through a focused assessment of completed, active, and pending clinical trials, our first aim in this review is to outline the scientific foundation, successes, and limitations of the BBBD strategies developed to date. Among 35 registered trials relevant to BBBD in neuro-oncology in the ClinicalTrials.gov database, mannitol was the most common drug-based method, followed by RMP-7 and regadenoson. MR-guided focused ultrasound was the most common device-based method, followed by MR-guided laser ablation, ultrasound, and transcranial magnetic stimulation. While most early-phase studies focusing on safety and tolerability have met stated objectives, advanced-phase studies focusing on survival differences and objective tumor response have been limited by heterogeneous populations and tumors, along with a lack of control arms. Based on shared challenges among all methods, our second objective is to discuss strategies for confirmation of BBBD, choice of systemic agent and drug design, alignment of BBBD method with real-world clinical workflow, and consideration of inadvertent toxicity associated with disrupting an evolutionarily-refined barrier. Finally, we conclude with a strategic proposal to approach future studies assessing BBBD.

## Introduction

The vertebrate blood-brain barrier (BBB) is a multi-faceted entity comprising metabolic, transport, and structural elements (1–6). Through strict regulation of the cerebral microenvironment, the BBB ensures optimal neuronal function (7). Because of this high-fidelity control mechanism, however, the BBB also presents a significant challenge in the management of brain tumors.

## The BBB Challenge in Neuro-Oncology

As one of the “success stories” in glioblastoma (GBM) treatment, one of the most common and fatal adult brain tumors, the highest tumor-to-blood concentration ratio achieved for temozolomide (TMZ) is <20%, largely due to the BBB (8–10). Although there is evidence of regional BBB disruption (BBBD) in all gliomas, much of the infiltrative component of the tumor is protected by intact BBB and this impermeability has been a key barrier to treatment success (11). Similar challenges have significantly impeded the pace of therapeutic advances for brain metastases, compared to the range of options available for systemic primaries, where immunotherapy-based regimens have had limited comparative success in advanced-phase clinical trials (12–16).

Attempts at overcoming the BBB, broadly categorizable into bypass or disruption methods, have spanned decades. Examples of the former include convection-enhanced drug delivery and direct implantation of carmustine-loaded polymers into the resection cavity, which have been comprehensively reviewed elsewhere (17–21). Another bypass strategy, ANG1005, is outlined in Supplementary Table 2 because it was developed more recently and is currently being investigated in multiple trials. In this review, however, we focus exclusively on BBBD strategies.

A search of the ClinicalTrials.gov database was conducted on June 2nd, 2019 for interventional studies assessing BBBD for brain tumors. Linked publications were assessed for detailed trial outcomes. We identified 35 total trials (25 completed, seven active, one terminated, one suspended, and one withdrawn; Supplementary Table 1). Twenty-six were drug-based and nine were device-based.

Our objective in this review is three-fold. First, we provide an overview of key trials pertinent to each drug- and device-based strategy, summarized below and outlined in more detail in Table 1. Subsequently, through a careful analysis of the methodologies used, we highlight several factors that we believe have hindered progress in this field thus far. These include definitive confirmation of BBBD, rationale for concomitant systemic drug choice, optimal integration into real-world clinical workflow, and the long-term toxicity associated with repeated BBBD. Finally, possible solutions to these issues and ways to increase chances of success in future trials are discussed.

**Table 1:** Overview of drug-based and device-based strategies for BBBD.

**Strategy**	**Type**	**BBBD duration**	**Systemic agent**	**Time to steady state**	**Molecular weight**	**Biological Rationale**	**Clinical Evidence**
Mannitol	Drug-based	15–18 min	Temozolomide	9 h	194.1 (g/mol)	- Osmotic BBBD shown to increase diffusion and bulk flow via endothelial cell shrinkage, widening of tight junctions, and vasodilation in rodents (22–25)	- Two Phase I studies; recurrent GBM; IA mannitol + bevacizumab or cetuximab: ° No DLTs (26, 27) ° Median PFS in those receiving bevacizumab was 10 months, longer than historical controls (28). - Phase II; brain metastases; methotrexate or carboplatin, IA mannitol (30-s infusion): median OS of 13.5 months compared with the historical survival of 2.3–4.2 months for the corresponding RPA classes of 2 and 3 (29).
RMP-7	Drug-based	2–20 min	Carboplatin	30 h (post-distribution)	371.2 (g/mol)	- Enhanced delivery of methotrexate and carboplatin and increased survival in tumor-bearing rats (30–32)	- Phase I; children with brainstem glioma, HGG, medulloblastoma/PNET, and ependymoma; IV carboplatin + RMP-7 (administered for 10 min beginning 5 min before the end of carboplatin infusion): ° No DLT (33) ° Subsequent Phase II: no objective response in patients with brainstem gliomas and HGGs; study was terminated for commercial reasons before first stage accrual goals could be met for other tumor strata (34). - Phase I; carboplatin + IV RMP-7 (given during the last 5 min of 15-min carboplatin infusion or as 10-min infusion starting 10 min after the completion of carboplatin): transient side effects related to carboplatin (35). - Two multicenter Phase II trials; adult HGG; IV RMP-7 + carboplatin: stable disease or partial or complete response (36). - Randomized double-blind placebo-controlled Phase II study; recurrent HGG: no difference between IV carboplatin + RMP-7 and carboplatin + placebo (37).
Regadenoson	Drug-based	30–180 min	Methotrexate	40–75 h	454.4 (g/mol)	- IV administration in rodents led to detection of 10 kDA dextrans that peaked at 30 min and lasted for 180 min. It also led to 60% higher brain TMZ levels in non-tumor-bearing rats (38, 39).	- Pilot: Adults with no intracranial disease undergoing regadenoson cardiac stress tests; FDA-approved dose of 400 μg: no BBBD observed using brain SPECT and CT imaging (40). - Pilot; recurrent GBM; IV regadenoson administered one hour after oral TMZ: no significant increase in TMZ levels as measured by microdialysis catheters (41).
MRgLA	Device	3–45 days	Bevacizumab	100 days	149a	- Thin laser probe (1.65–3.3 mm) stereotactically guided to target via diffusing tip (3–25 mm diameter) (42–44) - Hyperthermic ablation temperature of tumor core to 60–70°C; peritumoral region to 40–45°C	- NCT01851733 (Phase II): recurrent GBM patients; early (1-week post-LITT) or late (6-weeks post-LITT) IV doxorubicin injections (44) - K*^*trans*^* peaked immediately post-LITT followed by a decline over 4 weeks. Serum BSE levels peaked 1–3 weeks post-LITT and decreased to baseline by 6 weeks (44)
Cranial-implantable ultrasound (SonoCloud)	Device	<8 h	Cetuximab	114 h	146 kDa	- Small (11.5 mm diameter) implantable ultrasound transducer which introduces unfocused low-intensity sonication (1-MHz frequency) to area of interest (45–47). - Gas microbubbles (sulfur hexafluoride) facilitate BBBD for up to 8 h	- NCT02253212 (Phase I): recurrent GBM patients; dose-escalating carboplatin infusion; no permanent severe neurologic AEs occurred (48) - Adequate BBBD (11; 58%) patients vs. no/poor BBBD (PFS: 2.73 vs. 4.11 months, 95% CI, 0.11–0.94, *p* = 0.03; OS: 8.64 vs. 12.94 months, 95% CI, 0.16–1.14, *p* = 0.09) (48)
MRgFUS	Device	<4 h	Etoposide Doxorubicin	100–240 h 20–55 h	588.6 g/mol 543.5 g/mol	- Non-invasive ultrasound-based strategy used in conjunction with perfluorocarbon microbubbles. - Stable cavitation (oscillating without bursting) of microbubbles, followed by microstreaming (jet fluid around oscillating microbubbles) and extravasation of microbubbles across vessel walls due to tight junction disruption (49–52)	- NCT02343991 (Phase I): five patients with HGG; doxorubicin (one patient) or TMZ (four patients); no serious AEs were noted; 10–15% immediate increase in gadolinium enhancement with resolution ~20 h (53). - NCT03551249 (USA) and NCT03712293 (Korea): HGG patients - NCT03322813 (USA): Suspected infiltrative glioma on pre-operative brain imaging scans - NCT03714243 (Canada): Her2 positive breast metastasis

*AEs, adverse events; BBBD, blood-brain barrier disruption; CI, confidence interval; CT, computed tomography; DLT, dose-limiting toxicity; FDA, Food and Drug Administration; GBM, glioblastoma; HGG, high grade glioma; IA, intraarterial; IV, intravenous; LITT, laser interstitial thermal therapy; NCT, ClinicalTrials.gov identifier number; OS, overall survival; PNET, primitive neuroectodermal tumor; PFS, progression-free survival; RPA, recursive partitioning analysis; SPECT, single-photon emission computed tomography; TMZ, temozolamide*.

## Drug-Based Strategies

### Mannitol

The BBBD potential of hyperosmotic agents such as mannitol was first demonstrated in rodents in the 1970's (Table 1) (22–25). While numerous agents were initially tested, mannitol became the preferred choice owing to its already-established use in patients, and its ability to enhance survival in animal models led to the beginning of clinical investigations in the 1980's. Since then, mannitol-BBBD in patients has been reported in numerous trials (Table 1) (26–29, 54–62). Overall, Phase I and II testing has produced potentially encouraging results, with clinical safety well demonstrated and possible indications of effects on survival. However, control arms are lacking in all studies. Furthermore, proposals for overcoming the significant logistical challenges associated with mannitol administration in a clinical setting are scarce. Both of these issues need to be addressed in future investigations. Trials assessing mannitol combined with chemotherapeutic agents are continuing in one Phase I, four Phase I/II, and one Phase II trial as of the writing of this review, all of which are focused on high grade gliomas (HGGs) except one investigating primary CNS lymphoma. Five are single-arm and one is a non-randomized two-arm trial.

### RMP-7

Bradykinin is an endogenous compound that reversibly disrupts the BBB via endothelial B_2_ receptors and structural changes in tight junctions (63). RMP-7, a synthetic analog of bradykinin, induces similar changes but has a longer half-life and greater B_2_ receptor selectivity, and has been shown to increase drug delivery and survival in rodent models (Table 1) (30–32). Both intraarterial (IA) and intravenous (IV) administration of RMP-7 in combination with IV chemotherapy have been tested (33–37, 64–66). Early-phase testing of RMP-7 + carboplatin in HGG established clinical safety and showed potential efficacy, but these studies lacked control arms (33, 35). In contrast, a controlled Phase II trial reported no significant benefit of combining RMP-7 with carboplatin; dosing and timing of RMP-7 should be considered as possible issues (37). In the pediatric setting, a Phase I trial showed no dose-limiting toxicity (DLT) for RMP-7 + carboplatin but a subsequent Phase II trial was terminated for commercial reasons before the majority of first stage accrual goals could be met (Table 1) (34). RMP-7 is not currently being investigated for BBBD purposes.

### Regadenoson

Regadenoson is an adenosine A_2A_ receptor agonist FDA-approved for pharmacologic cardiac stress testing (67). Investigations into its BBBD potential began on the basis that adenosine is known to mediate BBB permeability, and regadenoson was shown to increase brain concentrations of various agents including TMZ in rats (Table 1), an effect likely mediated by decreased expression of tight junction proteins and vasodilation (38, 39). In two pilot studies, however, investigators failed to detect an increase in BBB permeability or evidence of enhanced TMZ delivery after regadenoson administration (Table 1) (40, 41). Dosing, timing, and method of BBBD detection are all potential reasons for this discrepancy. Although future studies employing different protocols may yield better results, there are currently no registered pending trials investigating this.

## Device-Based Strategies

### MR-gLA

Magnetic resonance-guided laser ablation (MRgLA) employs laser interstitial thermal therapy (LITT) to achieve cytoreduction and BBBD (42, 43). With a rational evaluation of LITT for BBBD, using brain-specific enolase (BSE) and K^*trans*^, a completed Phase II trial provides promising evidence in GBM patients with BBBD for 1-3 weeks (Table 1) (44). However, the capital and procedural cost of LITT, and the invasive nature of this procedure could be potential deterrents to widespread use (68). Furthermore, 1–3 weeks of BBBD may result in an unnecessarily prolonged exposure of healthy brain tissue to the external environment, which may reduce drug residence time by promoting leakage of drug back into the bloodstream. Although more Phase II testing can offer great insight into the efficacy of LITT for BBBD, only one ongoing Phase I trial, in pediatric glioma, is underway.

### Cranial Implantable Ultrasound

SonoCloud is an implantable transducer device which introduces unfocused low-intensity sonication to the area of interest, and has demonstrated BBBD in multiple preclinical studies (45–47). The safety and efficacy of SonoCloud is established in a Phase 1 trial, with a moderate increase in PFS and OS (Table 1) (48). However, despite being more amenable to repetitive sonication, implanting a permanent device increases the theoretical risk of infection. Another limitation involves the linear beam of the transducer and lack of beam focusing, making it difficult to assess the specificity of sonication in the region of interest. At this time, there are no active clinical trials investigating SonoCloud for BBBD.

### MRgFUS

**Figure 1:**
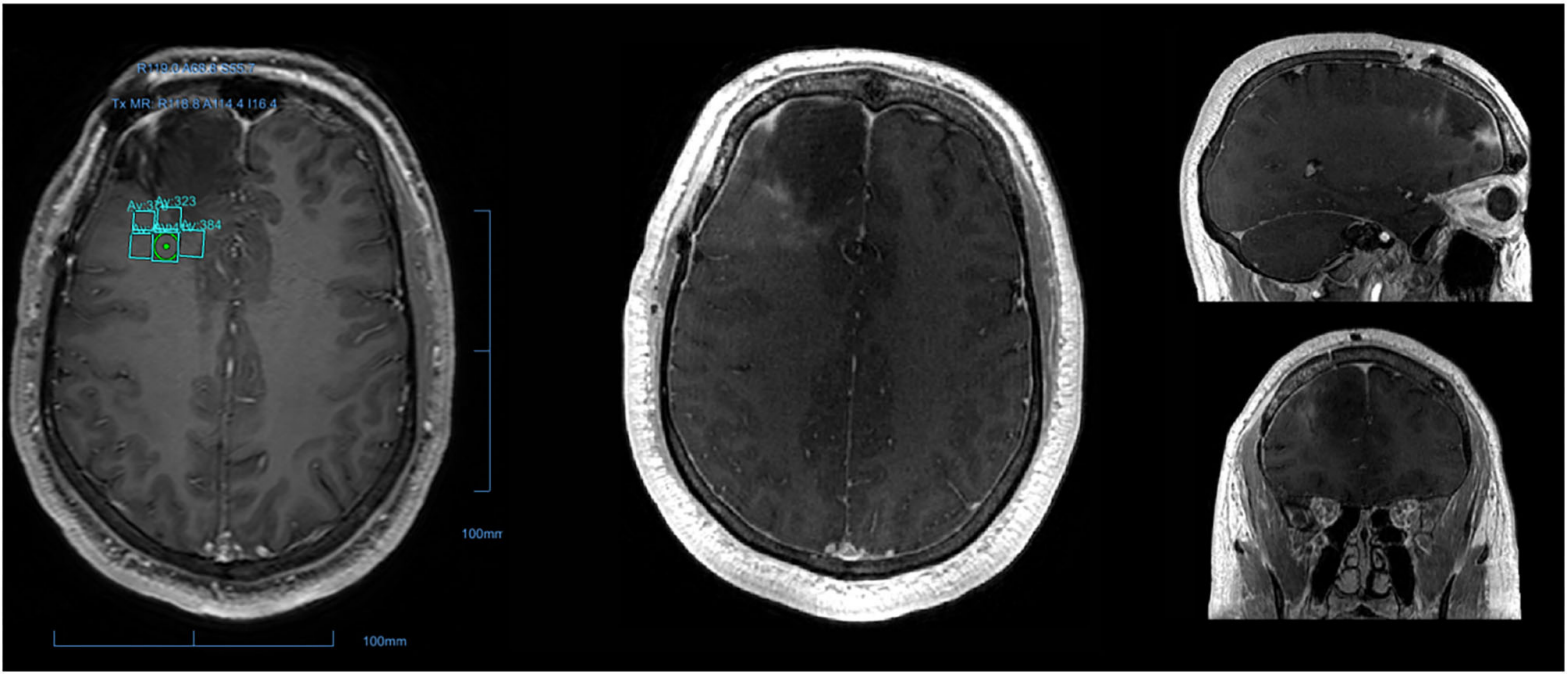
Post-operative peritumoral BBB disruption via MR-guided focused ultrasound. T1-weighted MRI from a patient who underwent surgery for glioblastoma followed by sonication of the resection boundary. The sonication field encompassed the 1 × 1 cm grid demonstrated, with nine sonication spots in each. Gadolinium, administered immediately after sonication was complete, was used to confirm precise disruption of the blood-brain barrier as indicated by the nine small areas of gadolinium leakage.

MR-guided focused ultrasound (MRgFUS) is a non-invasive ultrasound-based strategy for BBBD (49–52). A single-arm Phase I trial demonstrated BBBD by dynamic contrast-enhanced MRI (DCE-MRI) using MRgFUS in high-grade glioma (HGG) patients (53). Although BBBD was demonstrated in the completed Phase I trial by gadolinium enhancement seen on DCE-MRI, the extravasation of chemotherapy can only be accurately measured through serum biomarkers. However, one of the major advantages of MRgFUS is its precision, and that BBBD can be directly visualized to enable refinements in real-time rather than relying on a delayed assessment (Figure 1). From a safety standpoint, it eliminates the risks associated with implantation. In order to be successful for larger and/or diffusely infiltrating lesions, larger volumes of sonication and/or repeated sonication sessions would likely be required, necessitating repetitive shaving of the head and frame application, higher doses of microbubble administration, longer imaging time, or additional sessions, all with inherently increased risk to patients. MRgFUS is the most widely studied device based BBBD strategies with multiple non-randomized ongoing trials in Canada, USA, and Korea (Table 1).

## Challenges and Future Directions

The tremendous difficulty in overcoming the BBB for optimal delivery of therapeutics is reflected in the limited success of the decades of effort dedicated to this topic, and this remains a fundamental gap in research pertaining to the treatment of primary and secondary brain neoplasms. Consequently, one must approach testing BBBD methods in a stepwise manner to increase probability of ruling in or out the therapeutic utility of specific strategies.

One such approach involves go/no-go decision-making, in which specific criteria are used to make early decisions regarding continuing or stopping a drug development process (69). Simply put, the go/no-go system is a binary decision-aid in which two factors are required for a pass— “go” conditions must be met and “no go” criteria must fail—and can be used to make clinical investigations of BBBD strategies as efficient as possible.

Moving forward, the standardized go/no-go approach can help investigators identify which BBBD strategies should proceed with more thorough testing and which should be halted at earlier phases. Key challenges and considerations which need to be taken into account, and which we believe have received inadequate attention so far, are outlined below.

### Trial Design

One of the common limitations among studies published so far is the lack of control arms, applying equally to the drug- and device-based investigations reviewed here. Achieving balanced control arms in this setting is of course difficult and part of the broader challenge of designing effective randomized controlled trials (RCTs) in neurosurgery (70). Notwithstanding, simply incorporating arms without BBBD would enable investigators to make firmer conclusions early on regarding the potential of a given strategy. As an example, a controlled Phase II trial of RMP-7 + carboplatin produced conflicting results relative to preceding studies (37). Looking forward, very few of the pending trials include control arms, and improving on this front would expedite progress.

### Confirmation of BBBD

Serum and CSF markers of BBBD are potentially viable strategies for direct assessment of the success of BBBD methods. Brain-related proteins, such as S100B, monomeric transthyretin (TTR), and BSE, can be detected in the peripheral circulation in pathologies affecting BBB integrity (71). Assessment of CSF albumin is another potential method of assessing BBBD. Albumin is synthesized peripherally, is not catabolized within the CNS, and does not readily diffuse across an intact BBB (72). Aside from the invasive nature of this method, disruption of the blood-CSF barrier is not necessarily an accurate reflection of the status of the BBB.

In addition to serum and CSF biomarkers, there are also several imaging techniques used in assessing BBBD, including cerebral angiography, single-photon emission computed tomography (SPECT), static contrast-enhanced MRI, and DCE-MRI. Cerebral angiography is one of the oldest imaging techniques in neurosurgery. Assessment of changes in the blush pattern following administration of contrast can be used in assessing changes in BBB permeability; however, this is a relatively invasive technique. Another invasive technique is SPECT, a nuclear imaging scan that integrates computed tomography and a radioactive tracer. Challenges with SPECT imaging include the use of radiolabeled material and the time-sensitive nature of the results. Furthermore, the molecular size of the radiolabeled material, typically iodine or technetium, does not compare with most systemic therapeutic agents—the ultimate target of interest. Contrastingly, static contrast-enhanced MRI based on changes of gadolinium enhancement is relatively non-invasive in comparison to angiography and SPECT has often been used to assess BBB permeability in the modern imaging era. However, we advocate for DCE-MRI, in which several images are acquired in rapid succession enabling dynamic assessment of changes in contrast while also providing parameters pertinent to perfusion and permeability (73). Limitations of DCE-MRI, as stated in the MRgFUS discussion, include the spatial resolution and the ability to correlate dynamic variations in contrast enhancement with pharmacokinetic properties of test agents. Optimal sonication power has been established in previous studies (74, 75).

The timing of BBBD must align well with the pharmacokinetics of the agent. Furthermore, direct confirmation of drug accumulation and maintenance in the target tissue is critical. Direct drug measurement through microdialysis catheters is one option, though invasive. Alternatively, advances in drug development have facilitated the generation of radiolabeled agents for investigational use (76). This enables the possibility of assessing BBBD through non-invasive detection of the concentration of systemic agents of interest. However, this once again raises the need for pharmacological studies and complication of the regulatory process as the original identity of the compound has been altered—further illustrating the challenges involved in confirming BBB disruption.

### Choice of Systemic Agent

In-depth knowledge regarding the pharmacokinetic parameters of systemic agents, such as time to steady-state, is critical in optimizing the timing and duration of systemic agent delivery in relation to BBBD. Indeed, the time to steady-state of most systemic agents used in the reviewed trials was notably higher than the duration of BBBD. The success of cancer chemotherapy typically hinges upon circumventing dose-limited toxicity. In addition, pharmacokinetic properties of the drug must also be considered. Recent literature has proven that in order to improve brain exposure, polarity, and/or hydrogen-bonding capacity of the agent must be decreased (77). At the most basic level, these include interactions with drug-resistance factors such as extra-cellular receptors and intra-cellular DNA repair enzymes. At a deeper level, drug-related (e.g., tumor-binding specificity, low plasma clearance and lipophilicity) and tumor-related (e.g., size and mass effect exerted on surrounding brain) factors are also critical (78). Indeed, brain tumors influence their surrounding microenvironment, such that the established dynamics for an intact BBB are altered. Based on analyses of pharmacological properties of various compounds, it has been demonstrated that the distribution of high molecular weight compounds (such as monoclonal antibodies) is greater and faster in tumor tissue than normal brain, despite this being diffusion-limited (79). As such, it would be erroneous to assume uniform tumor cell vulnerability to drugs, as there are regional differences in BBB permeability and drug diffusion parameters.

These pharmacological considerations also put forth a predicament when designing a trial. The standard of care at the time of study initiation may not be the most effective drug against the target disease. Alternatively, a drug with suitable activity against the target disease may be unable to penetrate the BBB or the doses required for adequate tissue accumulation would be too toxic. This dilemma is readily applicable to the BBBD studies for GBM. For example, demonstration of increased permeability to carboplatin—a hydrophilic agent that does not readily penetrate the BBB—would in theory represent successful BBBD; however, the inferior effectiveness of this agent against malignant gliomas diminishes the relevance of the findings to the GBM population. Alternatively, increasing the concentration of TMZ—the current standard of care for GBM—in tumor tissue through an investigational BBBD method could be marked as a success.

### Applicability to Real-World Setting

In designing a BBBD strategy, unless the technique and its scheduling aligns with reasonable clinical care pathways and minimizes undue risk to patients, the uptake for the technique may be limited, even if shown to be successful.

One criticism of CNS drug development is that molecular targets have been the focus of research and the pharmaceutical industry without significant therapeutic advancements. The aim of early “go-no go” decisions is that investment in studies is targeted toward therapeutic potential, quickly ruling out ineffective mechanisms. The “go-no go” approach hinges on the approach that efficacious processes for data interpretation require frontload data interpretation. This requires that decision criteria is established during the design phase, and adoption of standard methodology (80). Thus, in the absence of measurability of drug function in the human brain an early decision is made not to pursue clinical efficacy trials (81). However, even after establishing the successful target molecular interaction, the researcher is faced with an additive layer of “go-no go” decision-making: is the demonstration of desired target molecular interaction sufficient to justify an efficacy trial? Or must one also require demonstration of functional brain effect? (82).

In the case of brain metastases, the optimal timing of BBBD in relation to treatments for the systemic primary must also be considered: should BBBD be included upfront and in conjunction with systemic therapies of established effectiveness (e.g., Herceptin for Her2 positive breast cancer), in the absence of evidence of metastases, or should this be isolated to patients with established metastases only? The case for upfront treatment is based on the rationale that if tumor metastasis is inevitable given the stage of the disease, systemic treatment with BBBD may be of utility in preventing or decreasing CNS burden. In such a case, drug-based strategies may offer benefit over more focused device-based ones. Alternatively, in the case of established brain metastases, a device-based strategy may enable focused therapeutic delivery to targets, in the brain-tumor interphase. For diffusely infiltrating gliomas, the target of sonication would be the FLAIR hyperintense regions where there is likely a high degree of tumor infiltration but an intact BBB. Also potentially necessitating targeted BBBD are cases in which a global increase in the systemic agent would introduce unacceptable toxicity, as has been demonstrated for TMZ in animal glioma models (83). Ultimately, both device- and drug-based strategies, once established, will likely have complementary roles.

### Toxicity

Although safety and tolerability within an early phase clinical trial setting has been shown for most BBBD methods, long-term analyses, and the effect of other concomitant medications on the neuronal environment when exposed to a more permeable BBB has not been studied in detail. The latter in particular is a concern that must be taken into consideration as we strive to incorporate BBBD from the research realm into the clinical sphere. Therefore, a comprehensive evaluation of the safety profile of many of the drugs currently in use for other disorders may be needed prior to wide-spread clinical implementation of a successful BBBD method.

## Conclusion

In this review of the landscape of BBBD, a variety of methods were discussed, with device-based strategies predominating the most recent time period. Early-phase trials have yielded encouraging results overall, but the lack of efficacy in later phase testing and termination of several lines of investigation indicate the urgent need for a more systematic, standardized approach to trial design in this space. The points raised here aim to generate an in-depth collaborative discussion in order to expedite progress in the development of effective BBBD strategies. The field of neuro-oncology is in need of a breakthrough.

## Author Contributions

All authors have contributed, read, and approved the final manuscript.

## Conflict of Interest

The authors declare that the research was conducted in the absence of any commercial or financial relationships that could be construed as a potential conflict of interest.
