# Supplementary material for: Blood-Brain Barrier Disruption in Neuro-Oncology: Strategies, Failures, and Challenges to Overcome
Source: Front Oncol. 2020 Sep 18;10:563840. doi: 10.3389/fonc.2020.563840 (PMC7531249; doi:10.3389/fonc.2020.563840)
Supplement: Supplementary file 1 [file Table_1.DOCX]

**Supplemental Table 1. Neuro-oncology clinical trials assessing BBBD strategies**

| NCT identifier / publication | Phase | Start and end of recruitment (year) | Country of study sponsor | Intervention(s) | Cohort of patients | | Sample size (Actual or estimated) | Primary Endpoint(s) | Result(s) |
| --- | --- | --- | --- | --- | --- | --- | --- | --- | --- |
| Device-based | | | | | | | | | |
| NCT03714243 | 1 | 2019 | Canada | **MRgFUS** | - Age 18-70 years (inclusive) with Her-2 positive breast cancer and brain metastases | | 10 | Rate of adverse events  Extent of BBB opening measured by degree of contrast enhancement on post-procedure MRI with contrast agent | Active |
| NCT02343991^83^ | 1 | 2015-2019 | Canada | **MRgFUS**, Doxorubicin (1 patient), Temozolomide (4 patients) | - Age 18-70 years (inclusive) with brain tumor that is <2.5cm in diameter | | 5 | Number of Device and Procedure related adverse events | -No adverse clinical or radiologic events related to the procedure  -BBB opening with an immediate 15-50% increased contrast enhancement and resolution 20 hours after |
| NCT03551249 | NA | 2018-Present | USA | **MRgFUS (ExAblate)** | - Age 18-80 years (inclusive) with glioblastoma with completed radiation/TMZ treatment | | 20 | Device and Procedure related adverse events | Active |
| NCT03322813 | NA | 2017-Present | USA | **MRgFUS (ExAblate)** | - Age 21-85 years (inclusive) with suspected infiltrative glioma on pre-operative brain imaging and planned surgical resection | | 15 | Device and Procedure related adverse events | Active |
| NCT03712293 | NA | 2018-Present | South Korea | **MRgFUS (ExAblate)** | - Age 19-80 years (inclusive) with glioblastoma with completed radiation/TMZ treatment | | 10 | Adverse events safety profile | Active |
| NCT02372409 | 1 | 2015- Present | USA | **MRgLA**, Doxorubicin, Etoposide | - **Arm A**: Newly-diagnosed glioma (Grade I-IV) in patients 3-21 years old (inclusive) who have had STR - **Arm B**: Recurrent brain tumor in patients 3-21 years old (inclusive) | | 12 | **Arm A:** 5-year PFS and OS  **Arm B:** 6-month PFS and 1-year QOL | Active |
| NCT01851733^71^ | 1 | 2013-Present | USA | **MRgLA**, Doxorubicin  **Arm A:** Historical controls  **Arm B:** at 6-8 weeks post MRgLA  **Arm C**: at 72 hours post MRgLA) | - Histologically confirmed GBM with current unequivocal progression based on MRI in patients ≥ 18 years old | | 45 | Vascular transfer constant (Ktrans), 6-month PFS of Arm B and C, as compared with historical controls | - K*^trans^* peaked immediately post-LITT followed by a decline over 4 weeks.  - Serum BSE levels peaked 1-3 weeks post-LITT and decreased to baseline by 6 weeks |
| NCT02253212^91^ | 1/2 | 2014- Present | France | **SONOCLOUD**, carboplatin | - Patients >18 years old with recurrent glioblastoma who have failed standard chemo-radiation therapy, tumor is smaller than 3.5 cm | | 20 | Safety of transient BBBD with SONOCLOUD^**^ | -BBBD was demonstrated through T1w MRI in 80% of sonications  -AEs included headache, cerebral edema, facial palsy, syncope. No carboplatin-related neurotoxicity  -BBBD was observed in 58% of patients, with PFS of 4.11 months and OS of 12.94 months  -No/poor BBBD (42%) patients had a median PFS of 2.73 months, and a median OS of 8.64 months |
| NCT02474966 | 2 | 2014-2015 | Italy | **Deep Trans-cranial Magnetic Stimulation** | - Histologically confirmed Grade IV glioma in patients 18-65 years who have undergone surgical resection at least 1 year prior to enrollment | | 15 | Change in BBB permeability based on the average value of the slope-value distribution function (CDF) obtained via DCE-MRI | Completed, results pending |
| Drug-based | | | | | | | | | |
| NCT00074178 | 2 | 2003-2006 | USA | **Osmotic BBBD***, Filgrastim, pegfilgrastim, cyclophosphamide, Cytarabine, dexamethasone, etoposide, methotrexate | | - Histologically or cytologically confirmed PCNSL in patients 16-75 years old | 22 | Clinical and radiographic 5-year PFS | Completed, results pending |
| NCT00253721 | 1 | 1998-2015 | USA | **Mannitol**, Melphalan | | - Histologically confirmed primary or metastatic PCNSL in patients ≥18 years old | 21 | MTD of Melphalan (defined as dose that results in Grade 3 toxicity in 33% of patients) as measured by NCI common toxicity criteria v2 toxicities | Terminated |
| NCT02571530 | 1 | 2015-Present | USA | **Mannitol**, single dose of Super-selective cerebral infusion of Herceptin | | - Patients with documented HER2/Neu+ breast cancer who are ≥ 18 years old | 48 | MTD of Herceptin | Active |
| NCT00397501 | 1/2 | 2013 | USA | **Mannitol**, methotrexate, carboplatin, sodium thiosulfate +/- Herceptin | | - Females 18-75 years old with clinical or radiological evidence of cerebral metastases from primary breast cancer and with known HER2 status | N/A | Safety and toxicity of regimen^**^ | Withdrawn |
| NCT00983398 | 1/2 | 2009- Present | USA | **Mannitol**, Melphalan, Carboplatin, and Sodium Thiosulfate | | - Patients 1-45 years of age with imaging/ histologically/ cytologically confirmed diagnosis of CNS embryonal tumor or germ cell tumor who have failed first-line therapy | 55 | MTD of intra-arterial melphalan given with intra-arterial carboplatin, osmotic BBBD and delayed IV sodium thiosulfate | Suspended |
| NCT00303849 | 1/2 | 2005-Present | USA | **Mannitol**, Etoposide,  melphalan, Carboplatin, and Sodium Thiosulfate | | - Patients 18-75 years old with histologically confirmed anaplastic oligodendroglioma or mixed glioma who are at least 28 days post TMZ therapy | 43 | MTD of melphalan^^^ (Phase I),  1-year PFS (Phase 2) | Active |
| Boockvar et al^10^ | 0/1 | 2009-2010 | USA | **Mannitol** + Super-selective Bevacizumab | | - Patients ≥18 years old with recurrent or progressive HGG following RT + TMZ, KPS> 60. Prior Bevacizumab allowed | 30  - Group 1 (Bev naïve): 19  - Group 2 (Prior Bev): 11 | MTD of bevacizumab (2 to 15mg/kg) following single session of osmotic BBBD with Mannitol followed by super-selective cerebral infusion of bevacizumab | - No dose-limiting toxicity  - Group 1 at 1 month: median of 46.9% reduction of enhancement volume; median of 32.1% reduction of perfusion  - Group 2 at 1 month: median 8.3% reduction of enhancement volume; median 25.5% reduction of perfusion |
| Fortin et al^12^ | 2 | 2005 | Canada | **Mannitol** + IV chemotherapy (methotrexate-based for lymphomas, carboplatin-based for others histologies) | | - Pediatric and adult patients with malignant brain tumors | 81 enrolled; 72 included in analysis | Radiographic response (MacDonald criteria) and overall survival | Overall median survival time from treatment initiation (months): GBM = 9.1; anaplastic oligodendroglioma = 13.9; metastases = 9.9 |
| Fortin et al^17^ | 2 | 1999-2005 | Canada | **IA Mannitol** + IA chemotherapy (methotrexate-based for lymphomas, carboplatin-based for others histologies) + etoposide + cyclophosphamide | | - Adults with histologically confirmed brain metastases, KPS> 50. Prior chemotherapy and RT allowed | 38  - Lung: 18  - Breast: 4  - Systemic lymphoma: 8  - Ovarian: 5  - Other: 3 | Overall survival | Median survival by histology:  - Lung: 13.5 months  - Breast: 8.1 months  - Systemic Lymphoma: 16.3 months  - Ovarian: 42.3 months |
| Doolittle et al^16^ | 0 | 1994-1997 | USA | **IA mannitol** + IA chemotherapy | | - Adult patients with malignant brain tumors | 221 | Safety and efficacy | Primary CNS lymphoma: 75% of patients achieved complete response.  All evaluable patients with primitive neuroectodermal tumor, metastatic disease, or germ cell tumors and 79% of evaluable patients with GBM achieved stable disease or better. |
| Burkhardt et al^11^ | 1 | 2009-2010 | USA | **Mannitol** + superselective intra-arterial cerebral infusion of bevacizumab | | - Adult patients with recurrent GBM | 14 | PFS and OS (long-term follow-up of phase I trial) | Median PFS: 10 months |
| Jahnke et al^18^ | 2 | 1981-2006 | USA | **Mannitol** + methotrexate- or carboplatin-based chemotheraphy | | - Pediatric and adult patients with embryonal and germ cell tumors | 54 | Toxicity, OS, and time to progression | Median OS was 2.8 years. No treatment-related deaths or neurological or vascular sequelae were observed |
| Angelov et al^20^ | 2 | 1982-2005 | USA and Israel | **IA mannitol** + methotrexate-based chemotherapy | | - Adult patients with newly diagnosed primary CNS lymphoma | 149 | Toxicity, PFS, and OS | Treatment was generally well tolerated. Median OS and PFS were 1.8 and 3.1 years, respectively. |
| Guilliame et al^21^ | 1 | 2005-2008 | USA | **Mannitol** + IA carboplatin, IA melphalan, and IV etoposide phosphate | | - Adult patients with refractory anaplastic oligodendroglioma or oligoastrocytoma | 13 | Toxicity and MTD | Treatment showed acceptable toxicity; MTD of IA melphalan was 4 mg/m^2^/day |
| Chakraborty et al^22^ | 1 | 2010-2015 | USA | **Mannitol** + superselective intra-arterial cerebral infusion of cituximab | | - Adult patients with recurrent GBM | 15 | Safety and MTD | No DLT associated with a cituximab of up to 250 mg/m^2^. Treatment was well tolerated overall. |
| Miyagami et al^43^ | N/A | Not stated (published 1990) | Japan | **Mannitol**, intra-carotid ACNU, radiation, resection | | - Patients 1-72 years old with grade III or IV astrocytoma, anaplastic oligodendroglioma, malignant ependymoma, malignant lymphoma, and germinoma | 21 | Toxicity, drug delivery to tumor, and therapeutic effect | 3-year survival among patients who received mannitol and ACNU was 67%. All but one of 5 patients who did not receive mannitol died within 1-6 months after surgery. |
| NCT02389738^92^ | 1 | 2015-2018 | USA | **Regadenoson**,  Temozolomide,  Microdialysis catheter | | - Patients age ≥18, confirmed diagnosis of recurrent HGG and a candidate for surgery | 5 | Change in AUC of temozolomide (TMZ) concentration (0-18 hours) in brain interstitium pre- and post- regadenoson infusion | Regadenoson did not result in a significant increase in TMZ concentration |
| Jackson 2017^46^ | 0 | 2017 | USA | **Regadenoson** | | - Healthy adult patients undergoing cardiac stress test | 12 | Change in brain penetration, distribution, and retention of Tc-99m and visipaque | No significant pre- to post-regadenoson differences were found. |
| Prados et al^36^ | 2 | 1995-1997 | USA | **RMP-7^$^,** carboplatin | | - Patients ≥ 16 years old with radiographically measurable recurrent malignant glioma on stable steroid dosing. Prior RT and chemo permitted | 122  RMP-7 + Carboplatin (62); placebo + Carboplatin (60) | Time to progression (Increase of ≥50% tumor volume, centrally reviewed OR clinical deterioration) | No statistically significant difference |
| Gregor et al^35^ | 2 | 1999 | USA | **RMP-7** | | - Adult patients with recurrent WHO grade III or IV AA or GBM | 92 recruited; 87 received treatment | Response rate | 79% of chemotherapy naïve patients and 24% of patients with prior chemotherapy showed either stable disease, partial response, or complete response. |
| Ford et al^34^ | 1 | 1998 | USA | **RMP-7**, carboplatin | | - Adult patients with recurrent HGG | 14 | Safety and tolerability | Side effects were generally transient. No evidence that RMP-7 altered the safety profile of carboplatin. |
| Warren et al^37^ | 1 | 2001 | USA | **RMP-7** | | - Childhood brain tumors | 25 | MTD of IV RMP-7 | Could not define MTD for lobradimil as dose-related toxicity was not observed |
| Cloughesy et al^63^ | 1 | (published 1999) |  | **RMP-7** (intra-arterial), carboplatin | | - Adults with recurrent malignant gliomas | 12 | Safety, tolerability, and preliminary efficacy of IA RMP-7 and carboplatin | No MTD was established and no grade 4 toxicities were observed. |
| NCT00005602^65^ | 1 | 2001-2005 | USA | **RMP-7**, radiation therapy, and carboplatin | | - Patients 3-21 years old with newly diagnosed brainstem gliomas | 13 enrolled; 12 fully evaluable for toxicity | Toxicity of RMP-7 and carboplatin delivered for 5 successive days during radiotherapy | Treatment for 3, 4, and 5 weeks was well tolerated. |
| NCT00019422^36^ | 2 | 1998-2003 | USA | **RMP-7**, carboplatin | | - Patients ≤21 years old with histologically-confirmed (except for brainstem gliomas), recurrent tumors (high and low grade glioma, medulloblastoma/ PNET, ependymoma), refractory to standard therapy | Enrolled: 41  Lobradimil + carboplatin: 40  Evaluated: 38 | Response rate and time to progression | No objective response in high grade gliomas and brainstem gliomas was observed. Study terminated for commercial reasons before first stage accrual goals could be met for other tumor strata |
| NCT00001502 | 1 | 1996-2008 | USA | **RMP-7**, carboplatin | | - Patients ≤21 years old with histologically-confirmed brain tumor that is either refractory to standard therapy or currently not eligible for any form of surgery/ chemotherapy/ radiation therapy | 30 | MTD of RMP-7 | Completed, results pending |

AA = anaplastic astrocytoma; ACNU = 3-(14-amino-2-methyl-5-pyrimidinyl)methyl)-1-(2-chloroethyl)-1-nitrosourea hydrochloride; EGFR = epidermal growth factor receptor; GBM = glioblastoma multiforme; HGG = high grade glioma; IA = intra-arterial; IV = intravenous; MTD = maximum tolerated dose; NSCLC = non-small cell lung cancer; PD = progressive disease; PFS = progression-free survival; PR = partial response; RT = radiotherapy; SD = stable disease; TMZ = temozolomide; WHO = World Health Organization

*The authors did not specify the exact agent and there are no publications linked to this trial

**Criteria for establishing safety not defined

^^^Defined as one dose level below the dose that produces grade 4 toxicity in 33% of patients, graded in accordance with National Cancer Institute Common Toxicity Criteria (NCI CTC) (version 3.0)

$RMP-7, Cereport, and Lobradimil are alternative names for the same bradykinin analogue
