# Supplementary material for: Blood-Brain Barrier Disruption in Neuro-Oncology: Strategies, Failures, and Challenges to Overcome
Source: Front Oncol. 2020 Sep 18;10:563840. doi: 10.3389/fonc.2020.563840 (PMC7531249; doi:10.3389/fonc.2020.563840)
Supplement: Supplementary file 2 [file Table_2.DOCX]

**Supplemental Table 2. Clinical trials based on ANG1005 – a BBB bypass strategy**

| NCT identifier / publication | Phase | Start and end of recruitment (year) | Country of study sponsor | Intervention(s) | Cohort of patients | Sample size (Actual or estimated) | Primary Endpoint(s) | Result(s) |
| --- | --- | --- | --- | --- | --- | --- | --- | --- |
| NCT01497665 | 2 | 2011-2015 | USA | **GRN1005**^^ (Angiochem) | - Patients ≥18 years old with at least 1 brain metastasis from histologically-confirmed NSCLC (known EGFR status) | 16 | Intra-cranial and extra-cranial response rate (RECIST v1.1 criteria) at 1-year | Terminated  PR: 2/10  SD: 3/10  PD: 5/10 |
| NCT02048059 | 2 | 2014-2017 | USA | **Ang1005**^^ (Angiochem) | - Patients ≥ 18 years old with at least 1 recurrent brain metastasis (≥0.5cm in diameter) from a breast primary | 72 | Intra-cranial response rate | Completed, results pending |
| NCT01480583 | 2 | 2011-2016 | USA | **GRN1005**^^ (Angiochem)  Arm 1: GRN1005 alone  Arm 2: GRN1005 + Herceptin | - Patients ≥ 18 years old with at least 1 recurrent brain metastasis (≥1.0cm in diameter) from a breast primary with known hormone status | 85 | Intra-cranial response rate | Completed, results pending |
| NCT03613181 | 3 | Estimated start: 2019 | USA | Arm 1: **ANG1005**  Arm 2: capecitabine, eribulin, or IV methotrexate | - HER2-negative breast cancer patients with newly diagnosed leptomeningeal disease and previously treated brain metastases | 150 (estimated) | Overall survival | Not yet recruiting |
| NCT01967810 | 2 | 2013-2017 | USA | Arm 1: **ANG1005** (bevacizumab-naïve recurrent GBM patients)  Arm 2: **ANG1005** and possible bevacizumab administered to bevacizumab-refractory recurrent GBM patients  Arm 3: **ANG1005** administered to recurrent WHO Gr. III anaplastic glioma patients | - Adult patients with recurrent HGG | 73 | Objective response rate (arms 1 and 3) and progression-free survival (arm 2) | Completed, results pending |
| NCT00539383^64^ | 1 | 2007-2010 | USA | **ANG1005** | - Adult patients with advanced solid tumors and metastatic brain cancer | 56 | Safety and tolerability; MTD | MTD was 650 mg/m^2^; ANG1005 was well tolerated and showed activity in pretreated patients |
| NCT00539344^60^ | 1 | 2007-2010 | USA | **ANG1005** | - Adult patients with recurrent WHO Grade II-IV glioma | 63 | Safety and tolerability; MTD | ANG1005 was well tolerated overall. MTD was 650 mg/m^2^. Dose-limiting toxicities included Grade 3 mucositis and Grade 4 neutropenia. |

^^ Both Ang1005 and GRN1005 are synthetic compounds composed of paclitaxel conjugated to Angiopep-2 which increases the efficacy of crossing the BBB by 100-fold
